# Phase-factor-dependent symmetries and quantum phases in a three-level cavity QED system

**DOI:** 10.1038/srep25192

**Published:** 2016-05-03

**Authors:** Jingtao Fan, Lixian Yu, Gang Chen, Suotang Jia

**Affiliations:** 1 State Key Laboratory of Quantum Optics and Quantum Optics Devices, Institute of Laser spectroscopy, Shanxi University, Taiyuan 030006, China; 2 Collaborative Innovation Center of Extreme Optics, Shanxi University, Taiyuan, Shanxi 030006, China; 3 Department of Physics, Shaoxing University, Shaoxing 312000, China

## Abstract

Unlike conventional two-level particles, three-level particles may support some unitary-invariant phase factors when they interact coherently with a single-mode quantized light field. To gain a better understanding of light-matter interaction, it is thus necessary to explore the phase-factor-dependent physics in such a system. In this report, we consider the collective interaction between degenerate V-type three-level particles and a single-mode quantized light field, whose different components are labeled by different phase factors. We mainly establish an important relation between the phase factors and the symmetry or symmetry-broken physics. Specifically, we find that the phase factors affect dramatically the system symmetry. When these symmetries are breaking separately, rich quantum phases emerge. Finally, we propose a possible scheme to experimentally probe the predicted physics of our model. Our work provides a way to explore phase-factor-induced nontrivial physics by introducing additional particle levels.

Symmetry and spontaneous symmetry breaking are central concepts in modern many-body physics^1^,^2^,^3^, due to their natural and clear relations with quantum phase transitions^4^. It is the emergence of a new phase that breaks an intrinsic symmetry of the system. More importantly, different symmetry-broken phases usually exhibit different ground-state properties. As a fundamental model of many-body physics, the Dicke model describes the collective interaction between two-level particles (such as atoms, molecules, and superconducting qubits, *etc*.) and a single-mode quantized light field^5^. In general, this model possesses a discrete *Z*_2_ symmetry. When increasing the collective coupling strength, this model exhibits a second-order quantum phase transition from a normal state to a superradiant state^6^,^7^,^8^,^9^,^10^,^11^,^12^,^13^,^14^,^15^,^16^,^17^,^18^,^19^,^20^,^21^,^22^, with the breaking of the discrete *Z*_2_ symmetry (Here we intentionally use the wording “normal/superradiant state” instead of “normal/superradiant phase”, since the word “phase” in the latter may be confused with another nomenclature “phase difference” which we will mention below). In the *Z*_2_-broken superradiant state, the ground state is doubly degenerate. In contrast, under the rotating-wave approximation, the Dicke model reduces to the Tavis-Cummings model^23^, with a continuous *U*(1) symmetry. In its corresponding *U*(1)-broken superradiant state, an infinitely-degenerate ground state can be anticipated. The above important symmetry and symmetry-broken physics of the Dicke and Tavis-Cummings models have been explored experimentally^24^,^25^,^26^,^27^,^28^,^29^. Recently, based on this two-level Dicke model, novel transitions between different symmetries^30^,^31^,^32^, especially from the discrete to the continuous^31^,^32^, have been revealed.

Notice that apart from its amplitude, the single-mode quantized light field *ε*_*φ*_ ≡ *ae*^*iφ*^ + *a*^†^*e*^−*iφ*^, where *a* and *a*^†^ are the corresponding annihilation and creation operators, has an important freedom of phase^33^,^34^,^35^ *φ*. In this sense, the interaction Hamiltonian of the standard two-level Dicke model becomes a phase-factor-dependent form, i.e.,


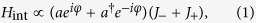


where *J*_*i*_ (*i* = ±) are the collective spin ladder operators. However, this phase factor *e*^*iφ*^ can be removed by a simple unitary transformation 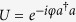. This means that the phase factor does not affect the system symmetries as well as the superradiance phase transitions^36^. So, it is a trivial variable in the standard two-level Dicke model. However, things become quite different when an extra energy level is introduced. In fact, when a three-level particle couples with a single-mode quantized light field via the electric dipole interaction, a nontrivial phase factor of light field can emerge naturally (see the supplementary material for a simple analysis). When the light-matter coupling strength becomes sufficiently strong, the rotating-wave approximation, under which any nontrivial phase factors can be removed by a certain unitary transformation (see the supplementary material for detailed discussions), breaks down, and thus new physics induced by the phase factor of the single-mode light field can be expected. In this sense, compared with the two-level systems, the three-level cavity QED systems serve as an ideal platform for studying physical effects induced by the phase factor of the quantized light field. Although some authors have considered interaction between three-level particles and the single-mode quantized light field^37^,^38^,^39^,^40^,^41^, these previous works ignored the potential appearance of the phase factor *e*^*iφ*^ (or equivalently, they just set *φ* = 0). That is, the physical effects induced by the phase factor *e*^*iφ*^ are still unknown. To gain a better understanding of light-matter interaction, it is thus necessary to explore the phase-factor-dependent physics in such a system.

In this report, we consider the collective interaction between degenerate V-type three-level particles and a single-mode light field, whose different components are labeled by different phases *φ*_1_ and *φ*_2_ [see the Hamiltonian (2) and Fig. 1 in the following]. Upon using this model, we mainly make a bridge between the phase difference, i.e., *ϕ* = *φ*_2_ − *φ*_1_, of the quantized light field and the system symmetry and symmetry broken physics. Specifically, when *ϕ* = *π*/2 or 3*π*/2, we find 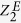 and 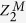 symmetries as well as a nontrivial *U*(1) symmetry. If these symmetries are broken separately, three quantum phases, including an electric superradiant state, a magnetic superradiant state, and a *U*(1) electromagnetic superradiant state, emerge. When *ϕ* = 0 or *π*, we reveal a *Z*_2_ symmetry and a trivial *U*^*tr*^(1) symmetry. When *ϕ* ≠ 0, *π*/2, *π*, and 3*π*/2, only the *Z*_2_ symmetry is found. If this *Z*_2_ symmetry is broken, we predict a *Z*_2_ electromagnetic superradiant state, in which both the electric and magnetic components of the quantized light field are collective excited and the ground state is doubly degenerate. Finally, we propose a possible scheme, in which the relative parameters can be tuned independently over a wide range, to probe the predicted physics of our model. Our work demonstrates that the additional particle level can highlight significant physics of the phase factor of the quadrature of the quantized light field, which can’t be captured in the two-level cavity QED systems.

## Results

### Model and Hamiltonian

**Figure 1:**
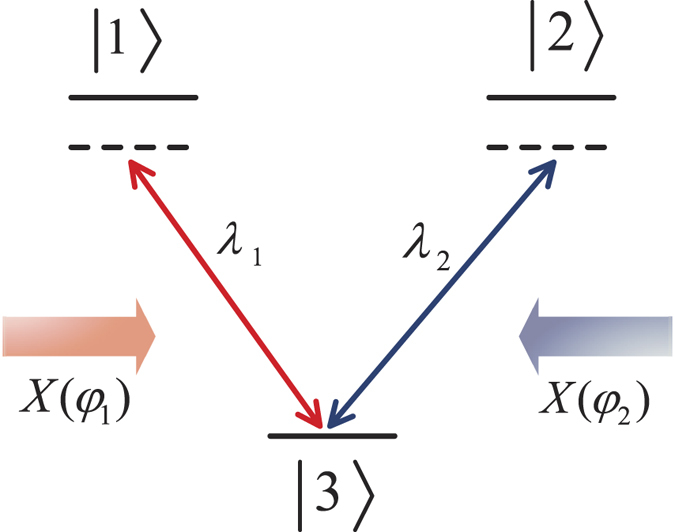
Schematic picture of our considered system. An ensemble of V-type three-level particles, in which 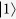 and 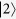 are degenerate, interacts with different phase-dependent components of a single-mode light field.

We consider *N* identical V-type three-level particles interacting with a single-mode quantized light field^37^,^38^, as sketched in Fig. 1. Each V-type particle consists of one ground state 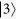 and two degenerate excited states 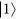 and 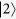. Two transitions 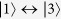 and 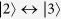 are governed by different phase-factor-dependent components of the quantized light field, respectively. In the absence of the rotating-wave approximation, the total Hamiltonian reads^37^


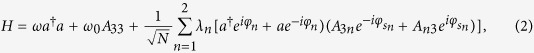


where *ω* is the frequency of the single-mode quantized light field, *ω*_0_ is the transition frequency between the ground state 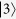 and the two degenerate excited states 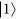 and 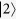, *λ*_*n*_ (*n* = 1, 2) are the collective coupling strengths, *φ*_*n*_ and 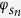 are the phases belonging to the quantized light field and the spin, respectively, and 
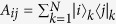
 (*i*, *j* = 1, 2, 3) represent the collective spin operators. Two sets of spin operators {(*A*_33_ − *A*_*nn*_)/2, *A*_3*n*_, *A*_*n*3_}(*n* = 1, 2) construct the *SU*(2) angular momentum algebra, respectively, i.e., [*A*_3*n*_, *A*_*n*3_] = *A*_33_ − *A*_*nn*_, [(*A*_33_ − *A*_*nn*_)/2, *A*_3*n*_] = *A*_3*n*_, and [(*A*_33_ − *A*_*nn*_)/2, *A*_*n*3_] = −*A*_*n*3_.

Generally speaking, the parametric space of the spin-boson model is a direct product of several subspaces^35^, i.e., 
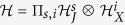
, where *J* and *X* label the spin and bosonic subspaces, respectively. To illustrate clearly this feature in our model, we rewrite the Hamiltonian (2) as a compact form


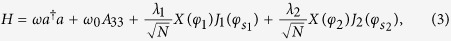


where *X*(*φ*_*m*_) = 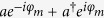 and 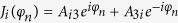 are the coordinates of the bosonic field and the spin in phase space^33^,^34^, respectively. Since 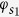 and 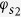 belong to two different spin subspaces 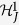 and 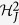, they are independent, and can be removed by a unitary transformation 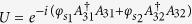. As a result, we set 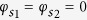 for simplicity. Whereas *φ*_1_ and *φ*_2_ belong to the same parametric space 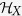, and cannot be removed simultaneously. In fact, there exists a unitary-invariant phase difference *ϕ* = *φ*_2_ − *φ*_1_, and thus we may directly set *φ*_1_ = 0 and *ϕ* = *φ*_2_. We emphasize that the phase factor *e*^*iϕ*^ of the quantized light field is a unique feature of our model. If the counter-rotating wave terms are negelected^38^,^39^,^40^,^41^ or two mode quantized light fields are considered^42^,^43^, the unitary-invariant phase factor *e*^*iϕ*^ disappears (see supplementary material for detailed discussions). As will be shown below, this phase factor *e*^*iϕ*^ plays an important role in determining symmetries and ground-state properties of the Hamiltonian (3).

### Symmetries

It is straightforward to find that the Hamiltonian (3) is invariant when performing the following transformation:


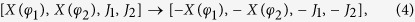


which indicates that the Hamiltonian (3) has a *Z*_2_ symmetry. In fact, when controlling *ϕ* as well as *λ*_1_ and *λ*_2_, the Hamiltonian (3) exhibits rich symmetries, as will be shown. For simplicity, we assume 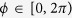 hereafter.

We first consider the case of *ϕ* = *π*/2 or 3*π*/2, in which the Hamiltonian (3) becomes


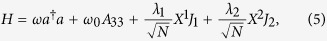


where *X*^1^ = *X*(0) and *X*^2^ = *X*(*ϕ*) are two quadratures of the quantized light field, which are called electric and magnetic components of the quantized light field, respectively. These couplings *X*^1^*J*_1_ and *X*^2^*J*_2_ support two different *Z*_2_ symmetries 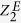 and 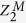, which can be broken separately^31^,^32^:


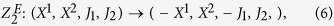



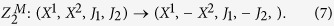


**Figure 2:**
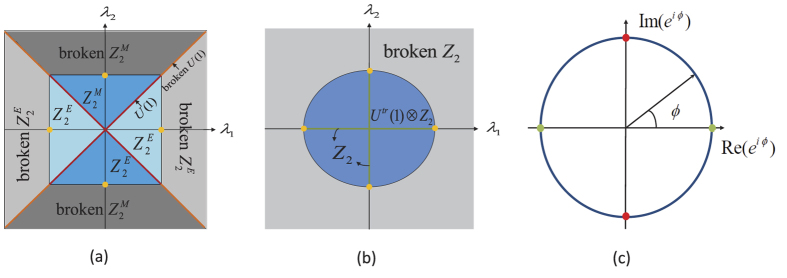
(**a,b**) Symmetric diagrams in the (*λ*_1_, *λ*_2_) plane and (**c**) symmetric properties for different *ϕ*. Parameters are chosen as (**a**) *ϕ* = *π*/2 or 3*π*/2, (**b**) *ϕ* = 0 or *π*, and (**c**) *λ*_1_ = *λ*_2_. In (**a**) and (**b**), the yellow points denote the critical point *λ*_*c*_ in Eq. (30). In (**c**), the big blue circle represents the *Z*_2_ symmetry. At the green and red points, the *U*^*tr*^(1) ⊗ *Z*_2_ and *U*(1) ⊗ 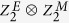 symmetries emerges, respectively.

More importantly, in the case of *λ*_1_ = *λ*_2_ = *λ*, the Hamiltonian (5) reduces to 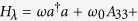

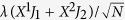
. For this Hamiltonian *H*_*λ*_, we find a conserved quantity


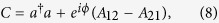


i.e., [*C*, *H*_*λ*_] = 0. In terms of this conserved quantity, we have *H*_*λ*_ = *e*^*iθC*^*H*_*λ*_*e*^−*iθC*^ (*θ* is an arbitrary real number), which implies that the Hamiltonian *H*_*λ*_ has a nontrivial *U*(1) symmetry (i.e., this *U*(1) symmetry can be broken by phase transitions), apart from the 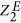 and 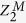 symmetries. We present an intuitive description of the symmetric properties of the Hamiltonian (5) in Fig. 2(a).

When *ϕ* = 0 or *π*, the Hamiltonian (3) is a simple sum of two standard Dicke models and exhibits a trivial *U*^*tr*^(1) symmetry, apart from the *Z*_2_ symmetry. To demonstrate those, we introduce two orthogonal states 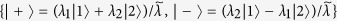 for *ϕ* = 0, and 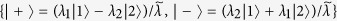 for *ϕ* = *π*. Taking account of these orthogonal states, we rewrite the Hamiltonian (3) as


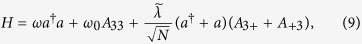


where 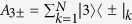 with 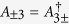 are the collective operators in the new basis, and 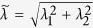 is an effective coupling strength. The Hamiltonian (9) shows clearly that the state 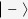 is completely decoupled from the system, and thus serves as a “dark state”. This dark state, which can be used to realize the coherent population trapping^44^,^45^, induces a trivial ground-state manifold. By introducing a unitary transformation 
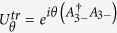
, we find 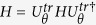, which indicates that the Hamiltonian (3) has a new *U*^*tr*^(1) symmetry. Because of the complete decoupling of the 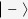 state, this *U*^*tr*^(1) symmetry can not be broken and is thus trivial. In Fig. 2(b), we give an intuitive description of these different symmetries.

When *ϕ* ≠ 0, *π*/2, *π*, and 3*π*/2, the operators *J*_1_ and *J*_2_ are coupled to two nonorthogonal components of the quantized light field, respectively. In this case, only the *Z*_2_ symmetry is found, and the predicted dark state is also absent.

From above discussions, it seems that the symmetries of the Hamiltonian (3) are sensitive to the phase difference *ϕ*, as shown in Fig. 2(c). The breaking of these symmetries are associated with rich quantum phases and their transitions, as will be discussed below (It should be noticed that the wording “symmetry breaking” refers to “spontaneous symmetry breaking” in this report, which is different from another nomenclature called “explicit symmetry breaking^46^”).

### Ground-state properties

To investigate quantum phases and their transitions, we need to consider ground-state properties of the Hamiltonian (2), which can be implemented by a generalized Holstein-Primakoff transformation^47^ and a boson expansion method^48^. In the case of three levels, we should apply the generalized Holstein-Primakoff transformation^37^,^38^,^42^,^43^, with a reference state called 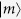, to rewrite the operators *A*_*ij*_ as


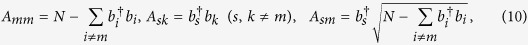


where 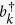 and *b*_*k*_ are the bosonic operators. For the Hamiltonian (2), we choose *m* = 3 to rewrite it as


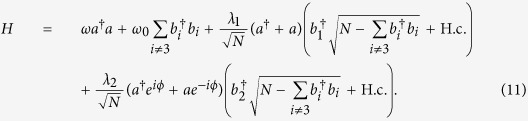


To explore the ground-state properties of the Hamiltonian (11) in the thermodynamic limit, we redefine these bosonic operators as


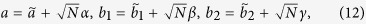


where *α* = *α*_1_ + *iα*_2_, *β* = *β*_1_ + *iβ*_2_, and *γ* = *γ*_1_ + *iγ*_2_. These complex auxiliary parameters 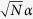, 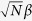, and 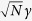 are the ground-state expectation values of the operators *a*, *b*_1_, and *b*_2_, respectively. Substituting Eq. (12) into the Hamiltonian (11) and then using the boson expansion method^48^, we obtain


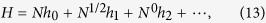


where


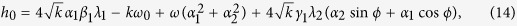


with 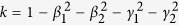, is the scaled ground-state energy. Based on Eq. (14), the scaled populations


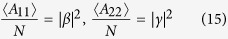


as well as the scaled mean-photon number


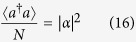


can be obtained by analyzing equilibrium equations ∂*h*_0_/∂*Y* = 0 (*Y* = *α*_1,2_, *β*_1,2_, *γ*_1,2_), i.e.,


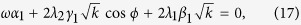



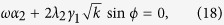



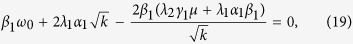



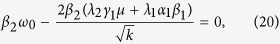



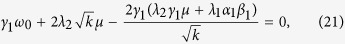



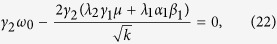


where *μ* = *α*_2_ sin *ϕ* + *α*_1_ cos *ϕ*. In addition, in order to distinguish the excitations of different components of the quantized light field, two extra quantities, including the scaled electric component of the quantized light field


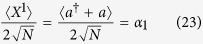


and the scaled magnetic component of the quantized light field


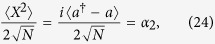


should be introduced.

In general, it is difficult to get a complete solution from the mean-field ground-state energy (14). In this report, however, we are able to analytically consider two specific cases discussed in the previous section, namely *ϕ* = *π*/2 and *λ*_1_ = *λ*_2_ = *λ*, to illustrate the crucial role of the phase factor *e*^*iϕ*^ in manipulating the ground state. Apart from their analytical solutions, another advantage of these two special choices is that they support typical symmetric properties of the system [see Fig. 2(a,c)], which signals the potential emergence of interesting symmetry-broken physics.

We first address the case of *ϕ* = *π*/2, in which the scaled ground-state energy in Eq. (14) turns into


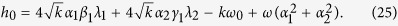


After a straightforward calculation, solutions of Eqs (17)–(22), are given by


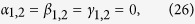



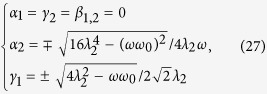



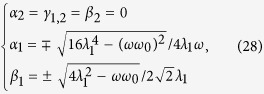



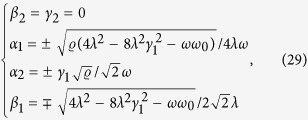


where 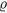  = 4*λ*^2^ + *ωω*_0_ and *λ* = *λ*_1_ = *λ*_2_ in Eq. (29).

By means of the stable condition (see Methods), we find that the solutions in Eqs (26)–(29), are stable in the following regions: (i) *λ*_1_ < *λ*_*c*_ and *λ*_2_ < *λ*_*c*_, (ii) 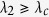 and 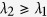, (iii) 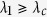 and 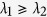, and (iv) 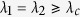, respectively, where


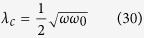


is a critical point.

**Figure 3:**
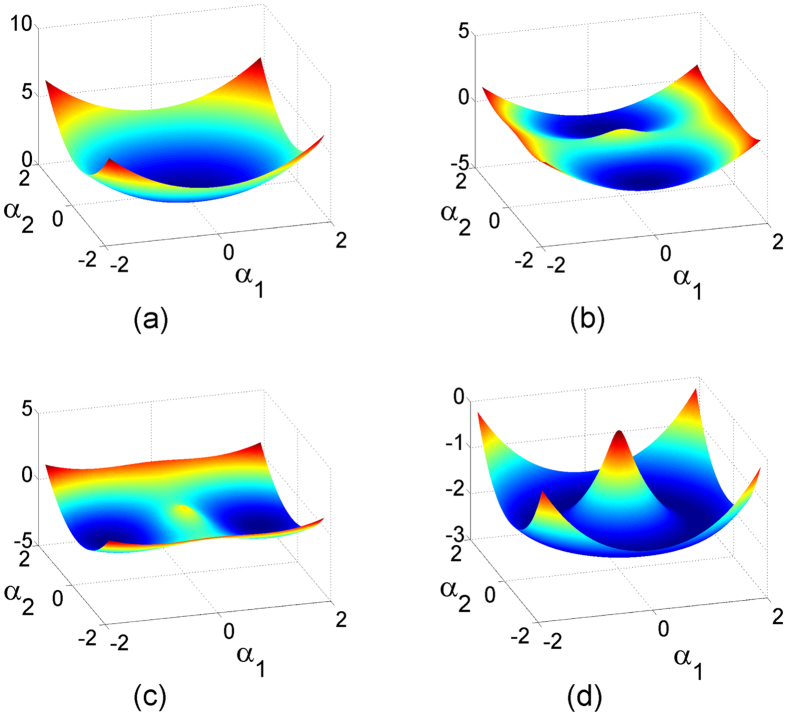
Energy surfaces *h*_0_ in the (*α*_1_, *α*_2_) plane. (**a**) *λ*_1_/*ω* = 0.2 and *λ*_2_/*ω* = 0.1, (**b**) *λ*_1_/*ω* = 0.7 and *λ*_2_/*ω* = 1.3, (**c**) *λ*_1_/*ω* = 1.3 and *λ*_2_/*ω* = 0.7, and (**d**) *λ*_1_/*ω* = *λ*_2_/*ω* = 1.3. The other parameters are chosen as *ω*_0_/*ω* = 1 and *ϕ* = *π*/2.

Considering both above corresponding solutions and the order parameters defined in Eqs (15), (16), (23), and (24), we reveal the following four quantum phases:

- *Normal state*. When *λ*_1_ < *λ*_*c*_ and *λ*_2_ < *λ*_*c*_, 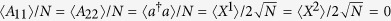. This means that no collective excitations occurs. In this phase, the ground-state manifold in the parametric space is a single point [see Fig. 3(a)], and the *Z*_2_ symmetry always exists.
- *Magnetic superradiant state*. When *λ*_2_ > *λ*_*c*_ and *λ*_2_ > *λ*_1_, 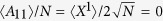, 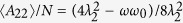, and 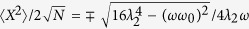. This means that the three-level particles in the 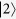 state and the magnetic component of the quantized light field are excited simultaneously, and the system has a doubly-degenerate ground state along the direction of the magnetic component of the quantized light field (i.e., the *α*_2_ axis) in the parametric space [see Fig. 3(b)]. In this quantum phase, the 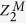 symmetry is broken.
- *Electric superradiant state*. When *λ*_1_ > *λ*_*c*_ and *λ*_1_ > *λ*_2_, 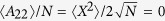, 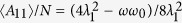, and 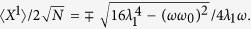 This means that the three-level particles in the 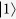 state and the electric component of the bosonic field are excited simultaneously, and the system has a doubly-degenerate ground state along the direction of the electric component of the quantized light field (i.e., the *α*_1_ axis) in the parametric space [see Fig. 3(c)]. In this quantum phase, the 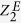 symmetry is broken.
- *U(1)* *electromagnetic superradiant state*. When *λ*_1_ = *λ*_2_ = *λ* > *λ*_*c*_, 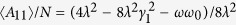, 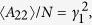 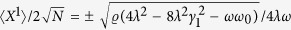, and 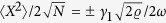. This means that the three-level particles in both the 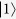 and 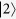 states as well as two quadratures of the quantized light field are excited simultaneously. Notice that 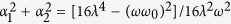 and 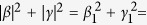 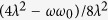, the ground-state manifold in the parametric space is thus a circular valley [see Fig. 3(d)], which signals the breaking of the nontrivial *U*(1) symmetry.

**Figure 4:**
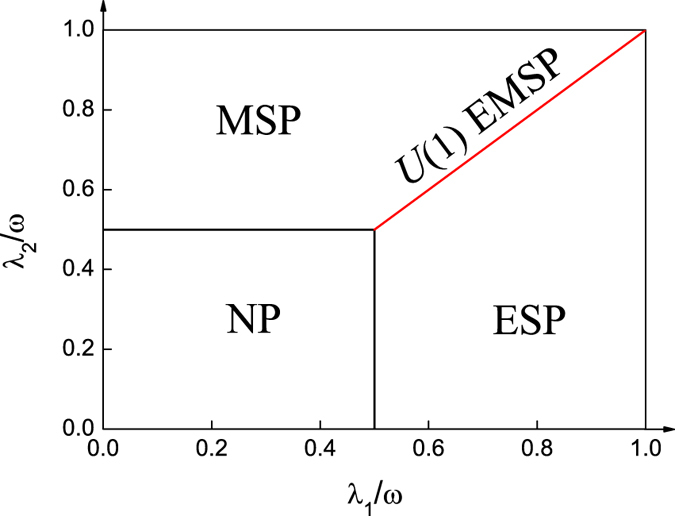
The ground-state phase diagram as a function of *λ*_1_ and *λ*_2_. In these abbreviations, NP denotes the normal state, ESP denotes the electric superradiant state, MSP denotes the magnetic superradiant state, and *U*(1) EMSP denotes the *U*(1) electromagnetic superradiant state (Red solid line). The other parameters are chosen as *ϕ* = *π*/2 and *ω* = *ω*_0_.

In Fig. 4, we plot the corresponding phase diagram as a function of *λ*_1_ and *λ*_2_. In terms of the scaled ground-state energy *h*_0_, we find that the transition from the normal state to the electric superradiant state or the magnetic superradiant state or the *U*(1) electromagnetic superradiant state is of second order. However, the transition from the electric superradiant state to the magnetic superradiant state is of first order. In addition, the results of *ϕ* = 3*π*/2 are the same as those of *ϕ* = *π*/2, and are thus not discussed here.

We now address the other case of *λ*_1_ = *λ*_2_ = *λ*, in which the ground-state energy in Eq. (14) turns into


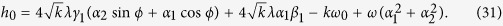


Following the previous procedure, the stable solutions of Eqs (26)–(29), should be divided into two cases, including (i) *ϕ* ≠ *π*/2 and 3*π*/2, and (ii) *ϕ* = *π*/2 or 3*π*/2.

When *ϕ* ≠ *π*/2 and 3*π*/2, we have


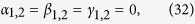


for *λ* < *λ*_*c*_(*ϕ*), and


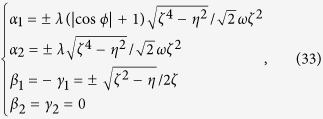


for 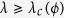, where


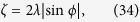



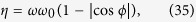


and


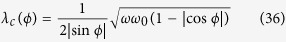


is a phase-dependent critical point. In the case of *ϕ* = *π*/2 or 3*π*/2, these stable solutions become


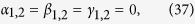


for *λ* < *λ*_*c*_(*ϕ*), and


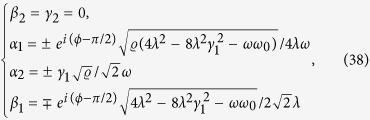


for 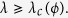


**Figure 5:**
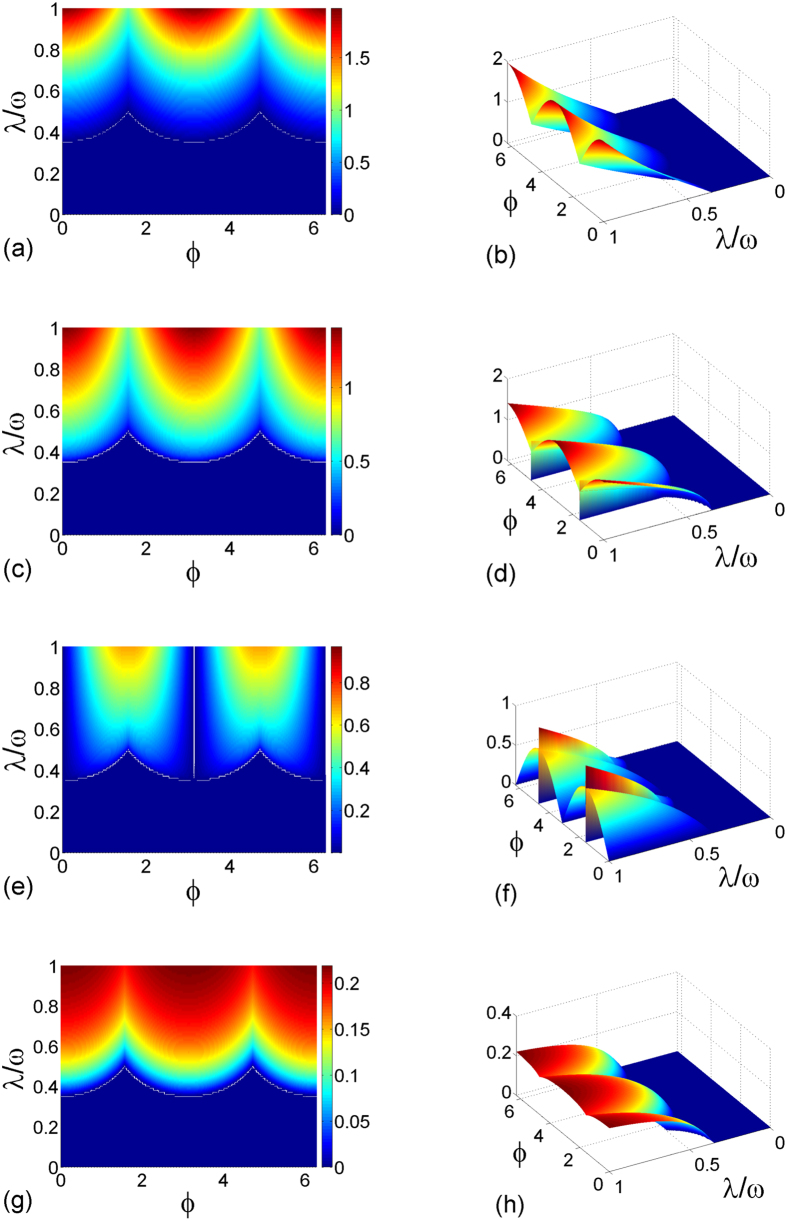
Four order parameters as functions of *λ* and *ϕ*. (**a,b**) 〈*a*^†^*a*〉/*N*, (**c,d**) 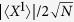, (**e,f**) 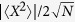, (**g,h**) 〈*A*_11_〉/*N*. The result of 〈*A*_22_〉/*N* is the same as that of 〈*A*_11_〉/*N*, and is thus not plotted here. The other parameter is chosen as *ω* = *ω*_0_.

The stable solutions in Eqs (32)–(38), govern the interesting phase-dependent ground-state properties. In Fig. 5, we plot four order parameters, 〈*a*^†^*a*〉/*N*, 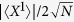, 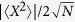, and 〈*A*_11_〉/*N*, as functions of *λ* and *ϕ*. This figure shows that when varying *ϕ* from 0 to 2*π*, these four order parameters as well as the critical point exhibit a periodic behavior, which is a manifestation of the competition between the electric and magnetic components of the quantized light field. In particular, when *ϕ* ≠ *π*/2 and 3*π*/2 with *λ* > *λ*_*c*_(*ϕ*), 〈*a*^†^*a*〉/*N* = |*α*|^2^ ≠ 0, and the superradiant state occurs. In this case, all of the nonzero parameters *α*_1,2_, *β*_1,2_ and *γ*_1,2_ have two feasible values, which means the breaking of the *Z*_2_ symmetry. When *ϕ* ≠ 0 and *π* in the *Z*_2_-broken superradiant state, both two quadratures of the quantized light field are excited [see Fig. 5(d,f)]. This indicates that a new phase, called the *Z*_2_ *electromagnetic superradiant state*, is predicted. A particularly interesting case is *ϕ* = *π*/2 or 3*π*/2 for *λ* > *λ*_*c*_(*ϕ*), in which the scaled electric component (magnetic component) of the quantized light field, i.e., 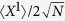 (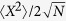), shows a nonanalytic behavior. As has discussed previously, under such a condition, the two quadratures of the quantized light field can acquire any available values continuously [see also Fig. 3(d)]. This is a definite signature of the breaking of the continuous *U*(1) symmetry and the *U*(1) electromagnetic superradiant phase thus emerges.

**Figure 6:**
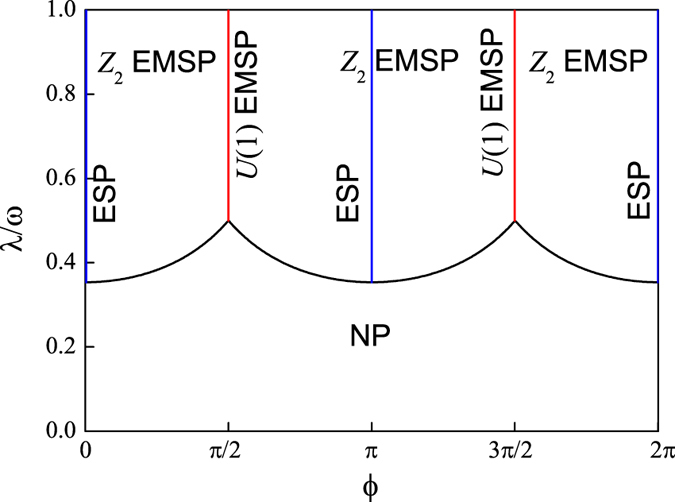
The ground-state phase diagram as a function of *λ* and *ϕ*. In these abbreviations, NP denotes the normal state, ESP denotes the electric superradiant state (Blue solid line), *Z*_2_ EMSP denotes the *Z*_2_ electromagnetic superradiant state, and *U*(1) EMSP denotes the *U*(1) electromagnetic superradiant state (Red solid line). The other parameter is chosen as *ω* = *ω*_0_.

In Fig. 6, we plot the corresponding phase diagram as a function of *λ* and *ϕ*. In terms of the scaled ground-state energy *h*_0_, we find that the transition from the normal state to the *Z*_2_ electromagnetic superradiant state is of second order. However, the transition from the *Z*_2_ electromagnetic superradiant state to the *U*(1) electromagnetic superradiant state is of first order.

**Figure 7:**
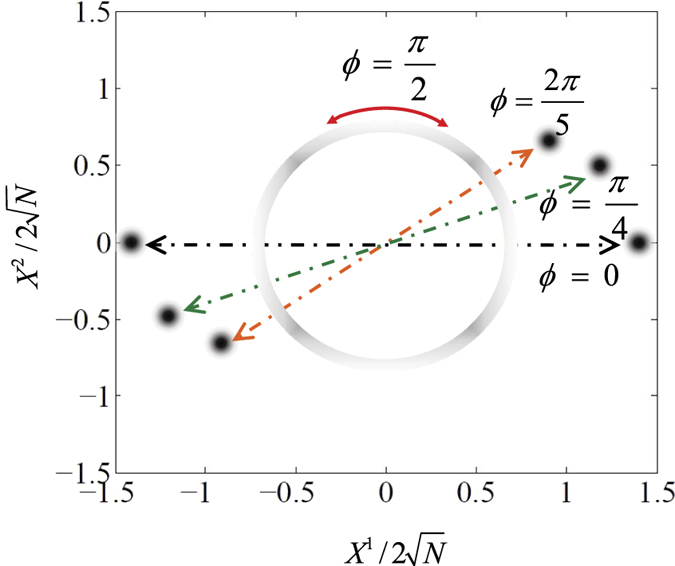
Schematic diagram of the ground-state distributions of the light field in phase space. If *ϕ* ≠ *π*/2 and 3*π*/2, 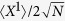 and 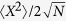 have two possible values, which is an intuitive manifestation of the breaking of the *Z*_2_ symmetry. However, if *ϕ* = *π*/2 or 3*π*/2, 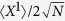 and 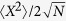 can be located at any point of a fixed circle. This, in contrast, signals the breaking of the *U*(1) symmetry. The other parameters are chosen as *ω* = *ω*_0_ = *λ*.

In the standard Dicke model, there is only one component of the quantized light field governing the system properties^8^. However, in our consideration, because of the existence of a finite phase difference, both two quadratures of the quantized light field contribute to the properties of the Hamiltonian (3). In fact, as shown in Fig. 7, the phase factor dramatically modulates the ground-state distributions of the quantized light field in its phase space, which give rise to a possibility of simultaneous excitations of 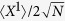 and 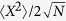.

### Possible experimental realization

The lack of experimentally-tunable parameters in the conventional three-level atoms prevents the direct observation of above predicted phenomena. Here we first propose a generalized balanced Raman channels^11^ to simulate this Hamiltonian, and then give a possible experimental implementation, based on the current experimental techniques of ultracold atoms in high-Q cavities^24^,^27^,^49^. This scheme has a distinct advantage that the corresponding parameters in the realized Hamiltonian can be independently tuned over a wide range.

**Figure 8:**
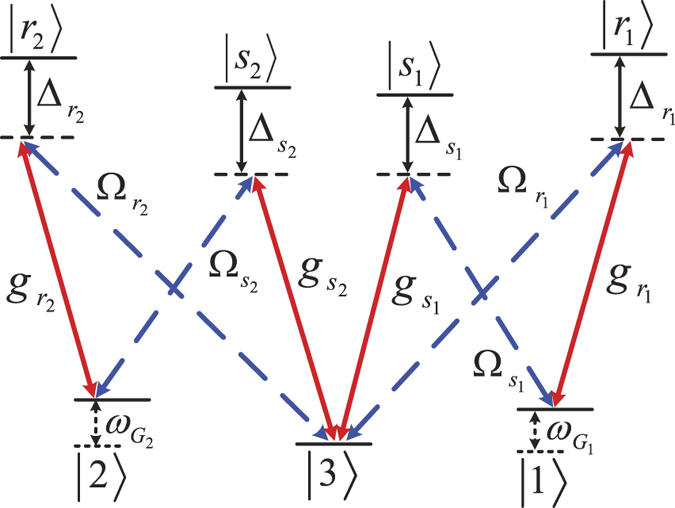
Atomic energy levels and their transitions.

As shown in Fig. 8, an ensemble of seven-level atoms is coupled with two pairs of Raman lasers and a single photon mode (i.e., quantized light field). Each atom has three ground states 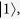 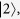 and 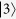, and four excited states 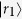, 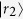, 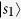, and 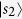. The photon mode mediates the transitions 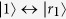, 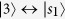, 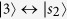, and 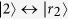 (red solid lines), with coupling strengths 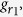 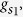 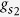, and 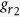, respectively. While two pairs of Raman lasers govern the other transitions 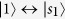, 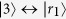, 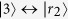, and 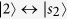 (blue dashed lines), with Rabi frequencies 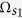, 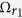, 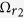, and 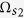, respectively. 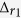, 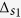, 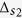, and 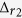 denote the detunings of the Raman lasers.

The total dynamics in Fig. 8 is governed by the following Hamiltonian:


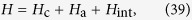


where


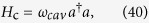



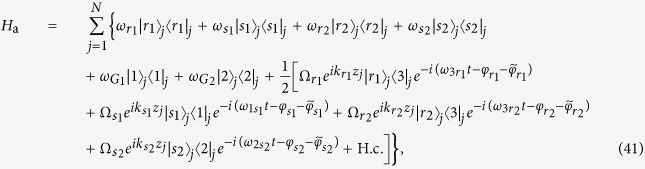



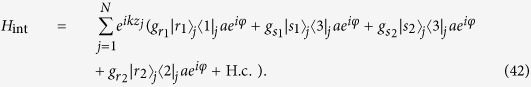


In the Hamiltonians (40)–(42), 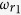, 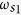, 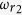, 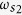, 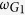, and 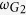 are the atomic frequencies, 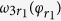, 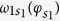, 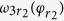, and 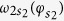 are the frequencies (initial phases) of two pairs of incident Raman lasers, *φ* is the phase of photon mode, *z*_*j*_ is the location of the *j*th atom in the laser beams, which support the wave numbers 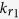, 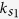, 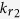, 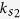, and *k* (note that 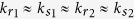), and phases 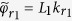, 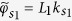, 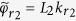, and 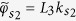 are acquired through three tunable optical lengths *L*_1_, *L*_2_, and *L*_3_.

In the interaction picture with respect to the free Hamiltonian 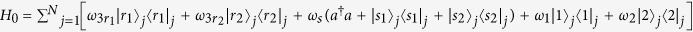, the Hamiltonian (39) is transformed as


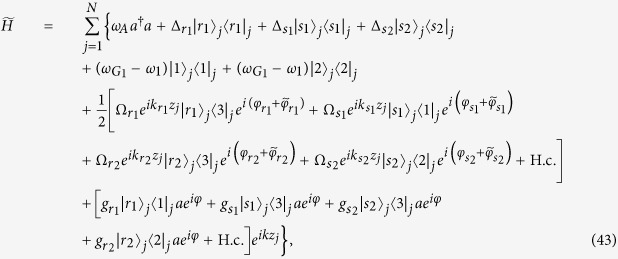


where 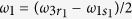, 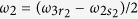, 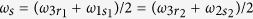, *ω*_*A*_ = *ω*_*cav*_ − *ω*_*s*_, 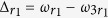, 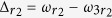, 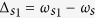, and 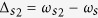.

In the large-detuning limit, i.e., 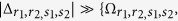 
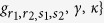
, where *γ* and *κ* are the atomic excited states’ linewidth and photon loss rate, respectively, we make an adiabatic approximation to eliminate all excited states of the Hamiltonian (43)^11^,^50^,^51^, and then obtain an effective Hamiltonian


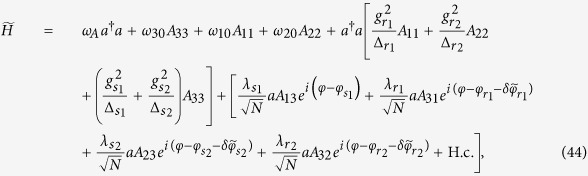


where 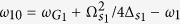, 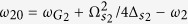, 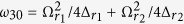, 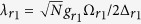, 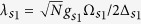, 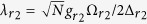, 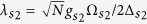, 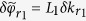, 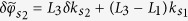, and 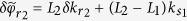, with 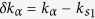 (*α* = *r*_1_, *r*_2_, *s*_2_).

When the parameters are chosen as 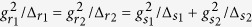, 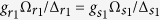, 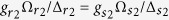, 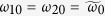, 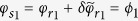, and 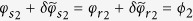, the Hamiltonian (44) reduces to


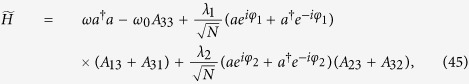


where


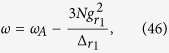



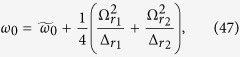



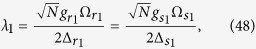



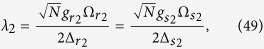



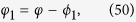



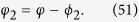


The Hamiltonian (45) is our required Hamiltonian. In this Hamiltonian, all parameters can be tuned independently. For example, *λ*_1_ and *λ*_2_ can be driven by the Rabi frequencies or detunings of Raman lasers^11^,^27^, and *φ*_1_ and *φ*_2_ can be individually tuned by adjusting the optical lengths *L*_1_, *L*_2_, and *L*_3_ or the wave-number difference *δk*_*α*_ (*α* = *r*_1_, *r*_2_, *s*_2_) of Raman lasers^52^.

**Figure 9:**
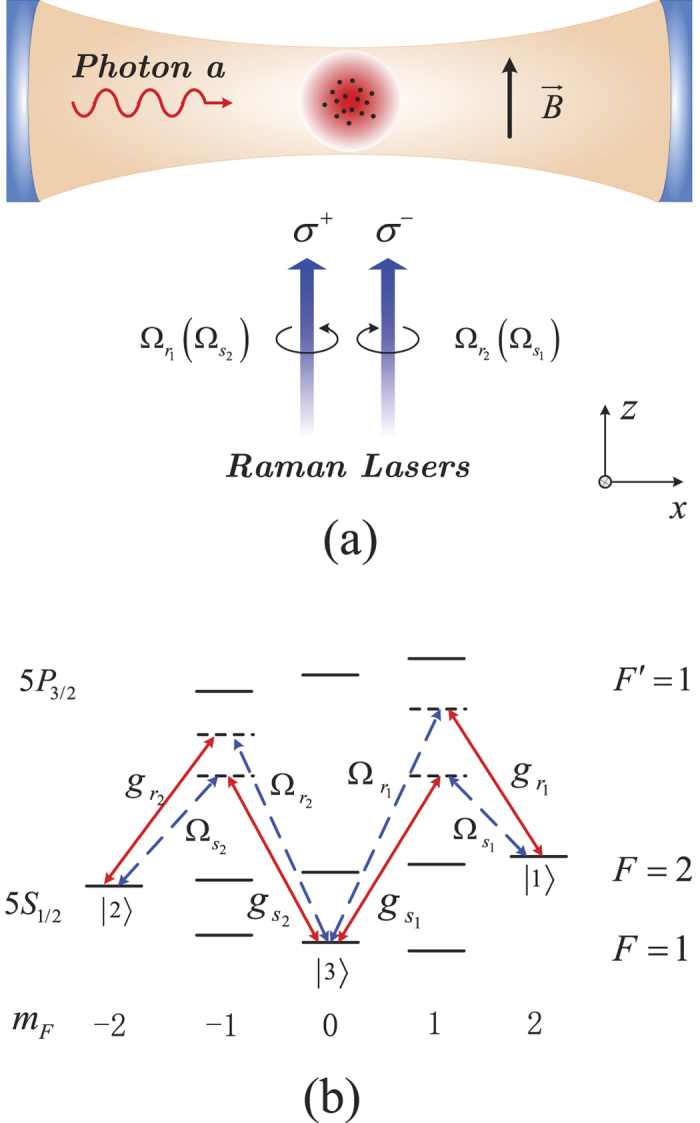
(**a**) The proposed experimental setup and (**b**) possible atomic excitation scheme based on the *D*_2_ line of ^87^Rb atom.

We now specify the implementation in an actual experimental setup. We consider an ensemble of ultracold atoms, loaded in a high-Q cavity, interacts simultaneously with a quantized cavity field and two pairs of Raman lasers. As shown in Fig. 9(a), a guided magnetic field *B* is applied along *z* direction to fix a quantized axis and split the Zeeman sublevels of the atomic ensemble, which confirms the distinct Raman channels. These two pairs of Raman lasers are right- and left-handed circularly polarized, respectively, and are assumed to co-propagate along the direction of the magnetic field. Moreover, the cavity field is linearly polarized along the *y* axis, which is perpendicular to the magnetic field. The detailed transitions are chosen as the *D*_2_ line of ^87^Rb atom, in which the three stable ground states 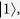 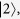 and 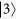 are chosen as some specific hyperfine sublevels of 5*S*_1/2_, such as 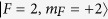, 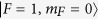, and 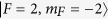, respectively, whereas the excited energy levels are assumed to be 5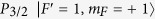 and 5*P*_3/2_ 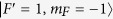 [see Fig. 9(b)].

Based on above energy levels and their transitions^53^, together with the current experimental conditions^27^, the atom-photon coupling strengths can reach 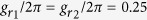 MHz and 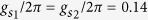 MHz, respectively. The atom number is set as^24^,^27^ 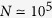. A proper choice of 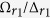 (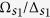), ranging from 0 to 0.04, is reasonable for the adiabatic condition in deriving Eq. (44). The practical parameters of the line width of cavity and atom are (*κ*/2*π*, *γ*/2*π*) = (0.07, 3.0) MHz^27^. Note that due to the far-detuning coupling, the spontaneous emission rate of the atomic excited states can be suppressed strongly by a factor of 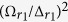 [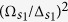]. Under these conditions, the collective coupling strength *λ*_1_ and *λ*_2_ can reach the order of several MHz, which is much large than the cavity and atomic decays, placing the system in a hamiltonian dynamics dominated regime. Furthermore, by properly tuning the frequencies of the cavity field and Raman lasers, it is not hard to achieve the superradiant condition 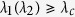.

Another issue to be specified is the experimental observation of different quantum phases, which lies in the measurement of the introduced order parameters, such as 〈*A*_11_〉, 〈*A*_22_〉, 〈*X*^1^〉, and 〈*X*^2^〉. In a practical experiment, for example, the atomic population 〈*A*_*ii*_〉 (*i* = 1, 2) can be straightforwardly obtained by detecting the transmission of certain probe field^54^,^55^, whereas the quadrature 〈*X*^*i*^〉 (*i* = 1, 2) could also in principle be measured by probing the cavity output using the technique of homodyne detection^56^.

The above proposal with ultracold atoms provides just one example of the potential experimental implementations. Recent advances in circuit QED system make it another alternative candidate^10^,^16^,^57^,^58^,^59^,^60^. We hope our report could stimulate related works in that area.

## Discussion

We briefly discuss the results of a deviation of the two levels 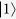 and 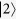. A deviation of the two levels just adds an extra term *δA*_*nn*_ (*n* = 1 or 2) to the Hamiltonian (2), where *δ* is the deviation. In such case, Eq. (4) still remains invariant, and the discrete *Z*_2_, 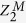, and 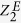 symmetries can thus emerge. That is, the *Z*_2_-broken phases can still be predicted. In contrast, due to existence of *δ*, no similar conserved quantity and decoupled state, as defined in the Result section, can be found. It implies that neither the trivial nor nontrivial *U*(1) continuous symmetries of the system can be found. Correspondingly, the *U*(1) electromagnetic superradiant state disappears.

Another point we should notice is that a thorough understanding of the Hamiltonian (2) demands paying more attention on the non-equilibrium properties. However, a complete description of the non-equilibrium features, which require more detailed and sophisticated analysis^14^,^15^, goes beyond the purpose of the present report. We leave this interesting problem for future investigation.

In summary, we have studied the V-type three-level particles, whose two degenerate levels are degenerate, interacting with a single-mode quantized light field, with a tunable *ϕ*. Upon using this model, we have made a bridge between the phase difference, i.e., *ϕ* = *φ*_2_ − *φ*_1_, of the light field and the system symmetry and symmetry broken physics. Specifically, when *ϕ* = *π*/2 or 3*π*/2, we have found 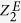 and 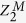 symmetries as well as a nontrivial *U*(1) symmetry. If these symmetries are broken separately, three quantum phases, including an electric superradiant state, a magnetic superradiant state, and a *U*(1) electromagnetic superradiant state, emerge. When *ϕ* = 0 or *π*, we have revealed a *Z*_2_ symmetry and a trivial *U*^*tr*^(1) symmetry. When *ϕ* ≠ 0, *π*/2, *π*, and 3*π*/2, only the *Z*_2_ symmetry can be found. If this *Z*_2_ symmetry is broken, we have predicted a *Z*_2_ electromagnetic superradiant state, in which both the electric and magnetic components of the quantized light field are collective excited and the ground state is doubly degenerate. Finally, we have proposed a possible scheme, in which the relative parameters can be tuned independently over a wide range, to probe the predicted physics of our introduced model. Our work demonstrates that the additional particle level can highlight significant physics of the phase factor of the quadrature of the quantized light field, which can’t be captured in the two-level cavity QED systems.

## Methods

To obtain stable quantum phases, we should introduce a 6 × 6 Hessian matrix 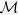, whose matrix elements can be calculated as 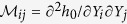 (*Y*_*i*_ = *α*_1,2_, *β*_1,2_, *γ*_1,2_). If the Hessian matrix 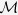 is positive definite (i.e., all eigenvalues of 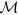 are positive), quantum phases are stable, and vice versa.

## Additional Information

**How to cite this article**: Fan, J. *et al*. Phase-factor-dependent symmetries and quantum phases in a three-level cavity QED system. *Sci. Rep*. **6**, 25192; doi: 10.1038/srep25192 (2016).

## Supplementary Material

Supplementary Information

## References

[b1] Huang K. Statistical Mechanics. (Wiley, New York, 1987).

[b2] Wen X. G. Quantum Field Theory of Many-body Systems: From The Origin of Sound To An Origin of Light and Electrons. (Oxford University Press, Oxford, 2004).

[b3] Sethna J. P. Statistical Mechanics: Entropy, Order Parameters, and Complexity. (Oxford University Press, New York, 2006).

[b4] Sachdev S. Quantum Phase Transitions. (Cambridge University Press, Cambridge, 1999).

[b5] Dicke R. H. Coherence in spontaneous radiation processes. Phys. Rev. 93, 99–110 (1954).

[b6] Wang Y. K. & Hioes F. T. Phase transition in the Dicke model of superradiance. Phys. Rev. A 7, 831–836 (1973).

[b7] Hioes F. T. Phase transitions in some generalized Dicke models of superradiance. Phys. Rev. A 8, 1440–1445 (1973).

[b8] Emary C. & Brandes T. Chaos and the quantum phase transition in the Dicke model. Phys. Rev. E 67, 066203 (2003). 10.1103/PhysRevE.67.066203 16241322

[b9] Chen G., Wang X., Liang J.-Q. & Wang Z. D. Exotic quantum phase transitions in a Bose-Einstein condensate coupled to an optical cavity. Phys. Rev. A 78, 023634 (2008).

[b10] Zhang Y. . Quantum phases in circuit QED with a superconducting qubit array. Sci. Rep. 4, 4083 (2014). 24522250 10.1038/srep04083 PMC3923215

[b11] Dimer F., Estienne B., Parkins A. S. & Carmichael H. J. Proposed realization of the Dicke-model quantum phase transition in an optical cavity QED system. Phys. Rev. A 75, 013804 (2007).

[b12] Larson J. & Lewenstein M. Dilute gas of ultracold two-level atoms inside a cavity: generalized Dicke model. New J. Phys. 11, 063027 (2009).

[b13] Nataf P. & Ciuti C. No-go theorem for superradiant quantum phase transitions in cavity QED and counter-example in circuit QED. Nat. Commun. 1, 72 (2010). 20842200 10.1038/ncomms1069

[b14] Nagy D., Kónya G., Szirmai G. & Domokos P. Dicke-model phase transition in the quantum motion of a Bose-Einstein condensate in an optical cavity. Phys. Rev. Lett. 104, 130401 (2010). 20481867 10.1103/PhysRevLett.104.130401

[b15] Keeling J., Bhaseen M. J. & Simons B. D. Collective dynamics of Bose-Einstein condensates in optical cavities. Phys. Rev. Lett. 105, 043001 (2010). 20867839 10.1103/PhysRevLett.105.043001

[b16] Viehmann O., von Delft J. & Marquardt F. Superradiant phase transitions and the standard description of circuit QED. Phys. Rev. Lett. 107, 113602 (2011). 22026666 10.1103/PhysRevLett.107.113602

[b17] Bakemeier L., Alvermann A. & Fehske H. Quantum phase transition in the Dicke model with critical and noncritical entanglement. Phys. Rev. A 85, 043821 (2012).

[b18] Bastidas V. M., Emary C., Regler B. & Brandes T. Nonequilibrium quantum phase transitions in the Dicke model. Phys. Rev. Lett. 108, 043003 (2012). 22400835 10.1103/PhysRevLett.108.043003

[b19] Emary C. & Brandes T. Phase transitions in generalized spin-boson (Dicke) models. Phys. Rev. A 69, 053804 (2004).

[b20] Lambert N., Chen. Y., Johansson R. & Nori F. Quantum chaos and critical behavior on a chip. Phys. Rev. B 80, 165308 (2009).

[b21] Wang T.-L. . Quantum Fisher information as a signature of the superradiant quantum phase transition. New J. Phys. 16, 063039 (2014).

[b22] Bhaseen M. J., Hohenadler M., Silver A. O. & Simons B. D. Polaritons and pairing phenomena in Bose-Hubbard mixtures. Phys. Rev. Lett. 102, 135301 (2009). 19392365 10.1103/PhysRevLett.102.135301

[b23] Tavis M. & Cummings F. W. Exact solution for an N-molecule-radiation-field Hamiltonian. Phys. Rev. 170, 379–384 (1968).

[b24] Baumann K., Guerlin C., Brennecke F. & Esslinger T. Dicke quantum phase transition with a superfluid gas in an optical cavity. Nature 464, 1301–1306 (2010). 20428162 10.1038/nature09009

[b25] Baumann K., Mottl R., Brennecke F. & Esslinger T. Exploring symmetry breaking at the Dicke quantum phase transition. Phys. Rev. Lett. 107, 140402 (2011). 22107178 10.1103/PhysRevLett.107.140402

[b26] Brennecke F. . Real-time observation of fluctuations at the driven-dissipative Dicke phase transition. Proc. Nat. Acad. Sci. USA 110(29), 11763 (2013). 23818599 10.1073/pnas.1306993110 PMC3718128

[b27] Baden M. P., Arnold K. J., Grimsmo A. L., Parkins S. & Barrett M. D. Realization of the Dicke model using cavity-assisted Raman transitions. Phys. Rev. Lett. 113, 020408 (2014). 25062149 10.1103/PhysRevLett.113.020408

[b28] Hamner C. . Dicke-type phase transition in a spin-orbit-coupled Bose–Einstein condensate. Nat. Commun. 5, 4023 (2014). 24895055 10.1038/ncomms5023

[b29] Feng M. . Exploring the quantum critical behaviour in a driven Tavis–Cummings circuit. Nat. Commun. 6, 7111 (2015). 25971985 10.1038/ncomms8111 PMC4479029

[b30] Nataf P., Baksic A. & Ciuti C. Double symmetry breaking and two-dimensional quantum phase diagram in spin-boson systems. Phys. Rev. A 86, 013832 (2012).

[b31] Baksic A. & Ciuti C. Controlling discrete and continuous symmetries in “superradiant” phase transitions with circuit QED systems. Phys. Rev. Lett. 112, 173601 (2014). 24836245 10.1103/PhysRevLett.112.173601

[b32] Fan J. . Hidden continuous symmetry and Nambu-Goldstone mode in a two-mode Dicke model. Phys. Rev. A 89, 023812 (2014).

[b33] Haroche S. & Raimond J. M. Exploring the Quantum: Atoms, Cavities, and Photons. (Oxford university press, Oxford, 2006).

[b34] Schleich W. P. Quantum Optics in Phase Space. (Wiley, Berlin, 2001).

[b35] Puri R. Mathematical Methods of Quantum Optics. (Springer, Berlin, 2001).

[b36] Xie Q. T., Cui S., Cao J. P., Amico L. & Fan H. Anisotropic Rabi model. Phys. Rev. X 4, 021046 (2014).

[b37] Baksic A., Nataf P. & Ciuti C. Superradiant phase transitions with three-level systems. Phys. Rev. A 87, 023813 (2013).

[b38] Cordero S., López–Peña R., Castaños O. & Nahmad–Achar E. Quantum phase transitions of three-level atoms interacting with a one-mode electromagnetic field. Phys. Rev. A 87, 023805 (2013).

[b39] Castaños O., Cordero S., López–Peña R. & Nahmad–Achar E. Single and collective regimes in three-level systems interacting with a one-mode electromagnetic field. Journal of Physics: Conference Series 512, 012006 (2014).

[b40] Cordero S., Castaños O., López–Peña R. & Nahmad–Achar E. A semi-classical versus quantum description of the ground state of three-level atoms interacting with a one-mode electromagnetic field. J. Phys. A: Math. Theor. 46, 505302 (2013).

[b41] Nahmad–Achar E., Cordero S., Castaños O. & López–Peña R. Phase transitions in three-level systems in a cavity. Phys. Scr. T160, 014033 (2014).

[b42] Hayn M., Emary C. & Brandes T. Superradiant phase transition in a model of three-level-systems interacting with two bosonic modes. Phys. Rev. A 86, 063822 (2012).

[b43] Hayn M., Emary C. & Brandes T. Phase transitions and dark-state physics in two-color superradiance. Phys. Rev. A 84, 053856 (2011).

[b44] Arimondo E. & Orriols G. Nonabsorbing atomic coherences by coherent two-photon transitions in a three-level optical pumping. Lettere al Nuovo Cimento della Societa Italiana di Fisica 17, 333–338 (1976).

[b45] Gray H. R., Whitley R. M. & Stroud C. R. Jr. Coherent trapping of atomic populations. Opt. Lett. 3, 218–220 (1978). 19684752 10.1364/ol.3.000218

[b46] Sinha D. & Amaratunga G. A. J. Electromagnetic radiation under explicit symmetry breaking. Phys. Rev. Lett. 114, 147701 (2015). 25910163 10.1103/PhysRevLett.114.147701

[b47] Holstein T. & Primakoff H. Field dependence of the intrinsic domain magnetization of a ferromagnet. Phys. Rev. 58, 1098–1113 (1949).

[b48] Klein A. & Marshalek E. R. Boson realizations of Lie algebras with applications to nuclear physics. Rev. Mod. Phys. 63, 375–558 (1991).

[b49] Landig R. . Quantum phases emerging from competing short- and long-range interactions in an optical lattice. arXiv: 1507.03500 (2015). 10.1038/nature17409 27064902

[b50] Ma J., Wang X., Sun C. P. & Nori F. Quantum spin squeezing. Phys. Rep. 509, 89–165 (2011).

[b51] Yu L. . Creating a giant and tunable spin squeezing via a time-dependent collective atom-photon coupling. Phys. Rev. A 89, 023838 (2014).

[b52] Zhan W. . Realization of two-dimensional spin-orbit coupling for Bose-Einstein condensates. arXiv: 1511. 08170 (2015). 10.1126/science.aaf6689 27846495

[b53] Steck D. A. “Rubidium 87 D line Data,” available online at http://steck.us/alkalidata (revision 2.1.4, 23 December 2010).

[b54] Chen Y.-C., Liao Y.-A., Hsu L. & Yu I. A. Simple technique for directly and accurately measuring the number of atoms in a magneto-optical trap. Phys. Rev. A 64, 031401 (2001).

[b55] Gibble K. E., Kasapi S. & Chu S. Improved magneto-optic trapping in a vapor cell. Opt. Lett. 17, 526–528 (1992). 19794547 10.1364/ol.17.000526

[b56] Wiseman H. M. & Milburn G. J. Quantum theory of field-quadrature measurements. Phys. Rev. A 47, 642–662 (1993). 9908961 10.1103/physreva.47.642

[b57] Manucharyan V. E., Koch J., Glazman L. I. & Devoret M. H. Fluxonium: Single cooper-pair circuit free of charge offsets. Science 326, 113–116 (2009). 19797655 10.1126/science.1175552

[b58] Teufel J. D. . Circuit cavity electromechanics in the strong-coupling regime. Nature 471, 204–208 (2011). 21390127 10.1038/nature09898

[b59] Xiang Z. L., Ashhab S., You J. Q. & Nori F. Hybrid quantum circuits: Superconducting circuits interacting with other quantum systems. Rev. Mod. Phys. 85, 623–653 (2014).

[b60] You J. Q. & Nori F. Superconducting circuits and quantum information. Physics Today 58(11), 42–47 (2005).

