# Supplementary material for: Phase-factor-dependent symmetries and quantum phases in a three-level cavity QED system
Source: Sci Rep. 2016 May 3;6:25192. doi: 10.1038/srep25192 (PMC4853727; doi:10.1038/srep25192)
Supplement: Supplementary Information [file srep25192-s1.pdf]

Phase-factor-dependent symmetries and quantum phases in a three-level cavity QED system

Jingtao Fan,[1,2] Lixian Yu,[3] Gang Chen,[1,2,*] and Suotang Jia[1,2]

[1]*State Key Laboratory of Quantum Optics and Quantum Optics Devices, Institute of Laser Spectroscopy, Shanxi University, Taiyuan 030006, China*

[2]*Collaborative Innovation Center of Extreme Optics, Shanxi University, Taiyuan, Shanxi 030006, China*

[3]*Department of Physics, Shaoxing University, Shaoxing 312000, China*

A. THE EMERGENCE OF NONTRIVIAL PHASE FACTORS FOR A THREE-LEVEL PARTICLE

Here we give a simple but general analysis of the electric dipole interaction between a three-level ($|1\rangle$, $|2\rangle$, and $|3\rangle$) particle and a single-mode light field, from which we can see the natural emergence of nontrivial phase factors of the light field. We assume the transitions $|1\rangle \longleftrightarrow |3\rangle$ and $|2\rangle \leftrightarrow |3\rangle$ are allowed, but $|1\rangle \longleftrightarrow |2\rangle$ is forbidden. The dipole form of the particle-field interaction Hamiltonian reads

$$H_{\text{int}} = -\mathbf{d}\cdot\mathbf{E}, \tag{S1}$$

where the dipole operator is defined as

$$\begin{aligned}\mathbf{d} &= \langle 3|\,\mathbf{d}\,|1\rangle\,|1\rangle\langle 3| + \langle 1|\,\mathbf{d}\,|3\rangle\,|3\rangle\langle 1| + \langle 2|\,\mathbf{d}\,|1\rangle\,|1\rangle\langle 2| + \langle 1|\,\mathbf{d}\,|2\rangle\,|2\rangle\langle 1| \\ &= \mathbf{d}_{31}\,|1\rangle\langle 3| + \mathbf{d}_{31}^{*}\,|3\rangle\langle 1| + \mathbf{d}_{21}\,|1\rangle\langle 2| + \mathbf{d}_{21}^{*}\,|2\rangle\langle 1|\,.\end{aligned} \tag{S2}$$

In addition, the light field is assumed as a single-mode plane wave, i.e.,

$$\mathbf{E} = \sqrt{\frac{\omega}{2\epsilon_0 V}}\left(\epsilon e^{ikr}a + \text{H.c.}\right), \tag{S3}$$

where ϵ is the complex polarization vector of the light field and a is the corresponding annihilation operator. Therefore, the Hamiltonian (S1) becomes

$$H_{\text{int}} = g_1\left(a\,|1\rangle\langle 3|\,e^{-i\varphi_a} + a\,|3\rangle\langle 1|\,e^{-i\varphi_b} + \text{H.c.}\right) + g_2\left(a\,|2\rangle\langle 3|\,e^{-i\varphi_c} + a\,|3\rangle\langle 2|\,e^{-i\varphi_d} + \text{H.c.}\right), \tag{S4}$$

where g_1 and g_2 are real atom-photon coupling strengths and we have defined

$$\begin{aligned}\epsilon\cdot\mathbf{d}_{31} &= g_1 e^{-i\varphi_a},\ \epsilon\cdot\mathbf{d}_{31}^{*} = g_1 e^{-i\varphi_b}, \\ \epsilon\cdot\mathbf{d}_{32} &= g_2 e^{-i\varphi_c},\ \epsilon\cdot\mathbf{d}_{32}^{*} = g_2 e^{-i\varphi_d}.\end{aligned} \tag{S5}$$

*Corresponding authors, e-mail: chengang971@163.com

The Hamiltonian (S4) shows that due to the complex nature of the polarization vector and dipole matrix elements, some phases, called φ_a, φ_b, φ_c, and φ_d, emerge. Furthermore, if the dipole matrix elements are assumed to be real, i.e., $\mathbf{d}_{31} = \mathbf{d}_{31}^*$ and $\mathbf{d}_{32} = \mathbf{d}_{32}^*$ (or equivalently, $\varphi_a = \varphi_b = \varphi_1$ and $\varphi_c = \varphi_d = \varphi_2$), the Hamiltonian (S4) turns into

$$H_{\rm int} = g_1(ae^{-i\varphi_1} + a^\dagger e^{i\varphi_1})(|1\rangle\langle 3| + |3\rangle\langle 1|) + g_2(ae^{-i\varphi_2} + a^\dagger e^{i\varphi_2})(|2\rangle\langle 3| + |3\rangle\langle 2|), \tag{S6}$$

which reduces to the same form as the Hamiltonian (2) of the main text.

B. V-TYPE THREE-LEVEL PARTICLES INTERACTING WITH TWO-MODE LIGHT FIELDS

When the states $|1\rangle$ and $|2\rangle$ of the V-type three-level particles are not degenerate, and moreover, interact with two light fields, as shown in Fig. S1(a), the corresponding Hamiltonian is given by

$$\begin{aligned} H = {} & \omega_1 a_1^\dagger a_1 + \omega_2 a_2^\dagger a_2 - \omega_{01} A_{11} + \omega_{02} A_{22} + \frac{\lambda_1}{\sqrt{N}}\left(a_1^\dagger e^{i\varphi_1} + a_1 e^{-i\varphi_1}\right)\left(A_{31} e^{-i\varphi_{s_1}} + A_{13} e^{i\varphi_{s_1}}\right) \\ & + \frac{\lambda_2}{\sqrt{N}}\left(a_2^\dagger e^{i\varphi_2} + a_2 e^{-i\varphi_2}\right)\left(A_{32} e^{-i\varphi_{s_2}} + A_{23} e^{i\varphi_{s_2}}\right), \end{aligned} \tag{S7}$$

where $a_1(a_1^\dagger)$ and $a_2(a_2^\dagger)$ are annihilation (creation) operators of two-mode bosonic fields, respectively. In contrast to the Hamiltonian (2) of the main text, the phases φ_1 and φ_2 in the Hamiltonian (S7) belong to two different parametric spaces. Therefore, all phases, including φ_1, φ_2, φ_{s_1}, and φ_{s_2}, can be removed by a unitary transformation

$$U_{\rm T} = e^{-i(\varphi_1 a_1^\dagger a_1 + \varphi_2 a_2^\dagger a_2 + \varphi_{s_1} A_{31}^\dagger A_{31} + \varphi_{s_2} A_{32}^\dagger A_{32})}. \tag{S8}$$

This means that these phases do not affect the symmetries and the ground-state properties of the Hamiltonian (S7), and thus we may directly set $\varphi_1 = \varphi_2 = \varphi_{s_1} = \varphi_{s_2} = 0$.

It was revealed that the Hamiltonian (S7) has three quantum phases, including a normal phase, a red superradiant phase (collective excitation of the light field a_1), and a blue superradiant phase (collective excitation of the light field a_2) [1]. Correspondingly, there exists two other Z_2 symmetries Z_2^R and Z_2^B, which can be broken separately:

$$Z_2^R : (a_1, a_2, A_{31}, A_{32}) \to (-a_1, a_2, -A_{31}, A_{32}), \tag{S9}$$

$$Z_2^B : (a_1, a_2, A_{31}, A_{32}) \to (a_1, -a_2, A_{31}, -A_{32}). \tag{S10}$$

The breaking of these symmetries are associated with different phase transitions (from the normal phase to the red superradiant phase or the blue superradiant phase). However, in the case of $\omega_1 = \omega_2$, $\omega_{01} = \omega_{02}$, and $\lambda_1 = \lambda_2$ [see Fig. S1(b)], we find a conserved quantity

$$C = i(a_1^\dagger a_2 - a_2^\dagger a_1) - i(A_{12} - A_{21}), \tag{S11}$$

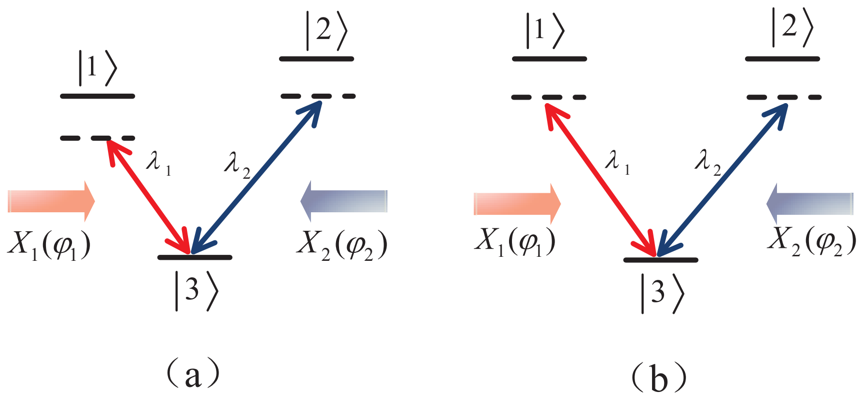

FIG. S1: (Color online) V-type three-level particles interacting with two-mode light fields. (a) Nondegenerate case ($\omega_1 \neq \omega_2$ and $\omega_{01} \neq \omega_{02}$), and (b) degenerate case ($\omega_1 = \omega_2$ and $\omega_{01} = \omega_{02}$).

which can induce a nontrivial $U(1)$ symmetry. Furthermore, when $\lambda_1 = \lambda_2 > \lambda_c$ in this degenerate case ($\omega_1 = \omega_2$ and $\omega_{01} = \omega_{02}$), this nontrivial $U(1)$ symmetry is broken, and a red and blue superradiant phase, with collective excitations of both the light fields a_1 and a_2, can thus be predicted.

C. RESULTS OF THE ROTATING-WAVE APPROXIMATION

Under the rotating-wave approximation, the Hamiltonian (2) of the main text becomes [2]

$$H_{\mathrm{R}} = \omega a^\dagger a - \omega_0 A_{33} + \frac{\lambda_1}{\sqrt{N}}\left[a^\dagger A_{31} e^{i(\varphi_1 - \varphi_{s_1})} + a A_{13} e^{-i(\varphi_1 - \varphi_{s_1})}\right] + \frac{\lambda_2}{\sqrt{N}}\left[a^\dagger A_{32} e^{i(\varphi_2 - \varphi_{s_2})} + a A_{23} e^{-i(\varphi_2 - \varphi_{s_2})}\right]. \tag{S12}$$

In contrast to the Hamiltonian (2) of the main text, all phases in the Hamiltonian (S12) can be easily removed by a unitary transformation

$$U_{\mathrm{R}} = e^{-i[(\varphi_1 - \varphi_{s_1})A_{31}^\dagger A_{31} + (\varphi_2 - \varphi_{s_2})A_{32}^\dagger A_{32}]}. \tag{S13}$$

Therefore, these phases are no longer effective parameters to control the system's symmetries.

In addition, for the Hamiltonian (S12), we can still introduce two new operators

$$\begin{cases} |+\rangle = \left[\lambda_1 |1\rangle e^{-i(\varphi_1 - \varphi_{s_1})} + \lambda_2 |2\rangle e^{-i(\varphi_2 - \varphi_{s_2})}\right] / \widetilde{\lambda} \\ |-\rangle = \left[\lambda_2 |1\rangle e^{-i(\varphi_1 - \varphi_{s_1})} - \lambda_1 |2\rangle e^{-i(\varphi_2 - \varphi_{s_2})}\right] / \widetilde{\lambda} \end{cases} \tag{S14}$$

to rewrite it as

$$H_R = \omega a^\dagger a - \omega_0 A_{33} + \frac{\widetilde{\lambda}}{\sqrt{N}}(a^\dagger A_{3+} + a A_{+3}). \tag{S15}$$

Clearly, the Hamiltonian (S15), which is a standard Tavis-Cummings model [3], shows that in the presence of the rotating-wave approximation, the symmetries and ground-state properties cannot be controlled by the phases of the

light field, although the dark state still exists. These results demonstrate that the counter-rotating terms are crucial to support a nontrivial phase difference.

---

[1] Hayn, M., Emary, C. & Brandes, T. Phase transitions and dark-state physics in two-color superradiance. *Phys. Rev. A* **84**, 053856 (2011).
[2] Cordero, S. López–Peña, R. Castaños, O. & Nahmad–Achar, E. Quantum phase transitions of three-level atoms interacting with a one-mode electromagnetic field. *Phys. Rev. A* **87**, 023805 (2013).
[3] Tavis M. & Cummings, F. W. Exact solution for an N-molecule-radiation-field Hamiltonian. *Phys. Rev.* **170**, 379-384 (1968).
